# Identification of bacterial contaminants in polyherbal medicines used for the treatment of tuberculosis in Amatole District of the Eastern Cape Province, South Africa, using rapid 16S rRNA technique

**DOI:** 10.1186/s41043-016-0064-y

**Published:** 2016-08-22

**Authors:** Elizabeth Bosede Famewo, Anna Maria Clarke, Anthony Jide Afolayan

**Affiliations:** Faculty of Science and Agriculture, University of Fort Hare, Alice, 5700 South Africa

**Keywords:** Polyherbal medicines, Tuberculosis, Bacteria, Public health, Sequencing

## Abstract

**Background:**

Polyherbal medicines are used for the treatment of many diseases in many African and Asian communities. With the increasing use of these remedies, several investigations have shown that they are associated with a broad variety of residues and contaminants. This study investigates the presence of bacteria in the polyherbal medicines used for the treatment of tuberculosis (TB) in the Eastern Cape Province of South Africa.

**Methods:**

Bacterial DNA was extracted from the polyherbal medicines, and a fragment of the bacterial 16S rRNA gene was amplified by PCR with universal primers 27F and 518R. The amplicons were visualised on agarose gel electrophoresis, followed by end repair and adaptor ligation. They were further purified and quantified using Library Preparation kit NEBNext® UltraT DNA Library Prep Kit for Illumina, and the amplicons were run on illumina’s MiSeq platform.

**Results:**

Different bacterial species were identified in all each of the polyherbal medicines. Generally, the most prominent and common bacteria recovered from all the samples were *Bacillus* sp., *Enterobacter* sp., *Klebsiella* sp., *Rahnella* sp., *Paenibacillus* sp., *Clostridium* sp. and *Pantoea* sp. Others are *Pseudomonas* sp., *Raoultella ornithinolytica*, *Salmonella enterica* and *Eubacterium moniliforme*.

**Conclusions:**

This study, thus, revealed the presence of pathogenic and non-pathogenic bacteria in the polyherbal medicines used for the treatment of tuberculosis in the study area. The implications of the findings are discussed in relation to the health care of the patients of tuberculosis in the study area, having in mind that they are immunocompromised individuals.

## Background

Polyherbal medicines have been used for various therapeutic purposes as far back as the origin of mankind. In South Africa, it is estimated that three million people currently use herbal remedies (polyherbal medicines) for their health care purposes especially in the treatment of diarrhoea, diabetes, stomach illnesses, wound infections and tuberculosis [1–3].

About 1 % of the South Africa population is estimated to develop tuberculosis yearly [4]. The country accounts for one quarter of the global burden of HIV-associated tuberculosis (TB) [5]. This is stimulated by the high rates of latent tuberculosis infection and the increase in the prevailing rates of infection [4]. In fact, about 50 % of people among the age group 15 and 77–89 % of adults have evidence of latent TB infection [6, 7]. The use of the current drug regimen combinations for TB is limited due to patients’ non-compliance, which has resulted in the rise of strains that are resistant to some or the entire first and second-line antibiotics [8]. The emergence of multi-drug resistant tuberculosis (MDRTB), extensively drug resistant tuberculosis (XDRTB), and totally drug resistant tuberculosis (TDR) has exacerbated the global health problem [9, 10]. Due to the high resistance of *M. tuberculosis* to commonly prescribed antimicrobials, relatively high cost and limited access to synthetically derived drugs, most communities especially in Africa still rely on the use of polyherbal medicine for the treatment of their ailments [11, 12]. Yet, these medicines have been reported to contain a number of microbial and heavy metal contaminants [13–16].

Medicinal herbs frequently harbour a large number of microbes originating from the soil, and these microorganisms normally adhered to leaves, stems, flowers, seeds and roots of plants [17]. The contamination of herbal products with Enterobacteriaceae, *Bacillus* spp., *Salmonella* spp*.*, S*taphylococcus aureus*, *Penicillium* spp. and *Aspergillus* spp. have been reported by [18]. Also, elevated levels of bacterial and fungal contaminants, such as *Escherichia coli*, yeast, *Penicillium* spp., *Aspergillus* and *Fusarium*, were observed in herbs and spices by [14, 19, 20]. The presence of these contaminants in the herbal products might adversely affect the health status of the consumers due to their immunocompromised conditions. Microbial infections have posed a health problem throughout the world, thus, the safety of the consumers of herbal products is of utmost importance.

To the best of our knowledge, the microorganisms present in some of the polyherbal medicines used for the treatment of TB in Amathole District Municipality of the Eastern Cape Province have not been investigated despite the mass consumption of the medicines. Most of these polyherbal remedies are prepared in the form of concoctions or infusions and are left at room temperature over a long period of time depending on how rapid the patients respond to treatment. During this period, the mineral elements present in these remedies may facilitate the growth of microorganisms. Thus, this study therefore aimed at identifying different bacteria present in some of the polyherbal medicines used for treatment of TB in the study area using molecular based technique.

## Methods

### Sample collection

**Fig. 1:**
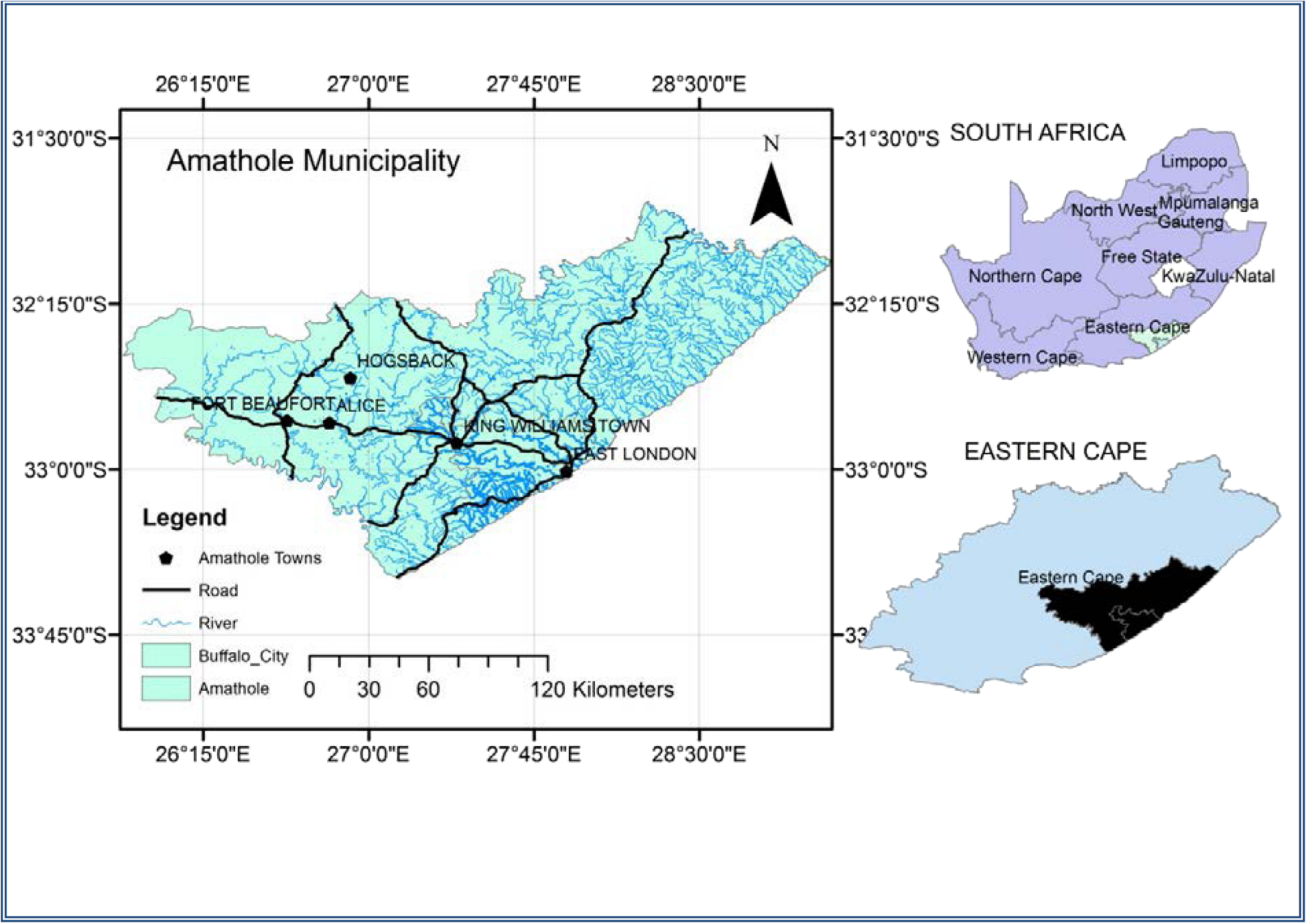
Map of Amathole District Municipality

A total of nine polyherbal medicines used for the treatment of TB were purchased from the traditional herbal sellers in five communities, namely East London (EL), King Williams Town (KWT), Hogsback (HB), Alice (AL) and Fort Beaufort (FB) as shown in Fig. 1. Each remedy was labelled and coded according to the place of collection, viz: King Williams Town site A (KWTa), King Williams Town site B (KWTb), King Williams Town site C (KWTc), Hogsback first site (HBfs), Hogsback second site (HBss), Hogsback third site (HBts), East London (EL), Alice (AL) and Fort Beaufort (FB). The small number of remedies obtained in this study was due to the fact that only a few traditional healers treat and sell the remedies for TB. They claim to have acquired the knowledge from their ancestors, and this knowledge is been transferred from one generation to another. The samples were then transported to Medicinal Plants and Economic Development (MPED) Research Centre for analysis.

### DNA extraction

The total bacterial DNA was extracted in a clean and sterilized environment using ZR Fungal/Bacterial MiniPrep™ Kit (Zymo Research, USA). The method of [21] with slight modification was used for the extraction. One millilitre of each sample was pipetted into sterile eppendorff tubes and centrifuged at 12,500 rpm for 10 min. The supernatant was discarded, and the cell pellets were collected. The protocol in the extraction kit was followed.

### PCR amplification of bacterial DNA

Polymerase chain reaction (PCR) was performed using the extracted DNA from each of the samples. The bacterial 16S rRNAs were amplified using the oligonucleotide primers 27F (5′-GGT AGA GTT TGA TCC TGG CTC AG-3′) and 518R (5′-ATT ACC GCG GCT GCT GG-3′). The 16S rRNA gene contains nine variable regions (designated V1 to V9) of which we chose the V1-V3 regions, which has previously proven useful in research-oriented metagenomic surveys [22]. A total reaction volume of 25 μL was used, which contained 12.5 μL Master Mix (Thermo Scientific, EU Lithuania), 1 μL each of 10 μM of both forward and reverse primer solutions (Inqaba Biotech, SA), 5.5 μL of nuclease free water and 5 μL template DNA. Reactions was performed in the thermocycler (Bio-Rad Mycycler, USA) using the following cycling conditions: initial denaturation at 94 °C for 1 min, followed by 30 cycles of denaturation at 94 °C for 1 min, annealing at 58 °C for 1 min, extension at 72 °C for 1.5 min and final extension at 72 °C for 10 min [23]. In order to confirm the products size, 5 μL of the amplicons was analysed by gel electrophoresis in 1 % agarose (Merck, SA) stained with 3 μL ethidium bromide (Sigma-Aldrich, USA). A 100 bp DNA ladder for 16S rRNA (Thermo Scientific, (EU) Lithuania) was included for band size estimation purposes. All gels were run in 0.5X TBE buffer at 95 V for 1 h and visualised by UV trans-illumination (Alliance 4.7, France).

### Purification of 16S rRNA gene amplicons and sequencing

The bacterial 16S rRNA gene amplicons were purified with the Zymoclean™ Gel DNA Recovery kit (ZymoResearch Corporation, Irvine, USA) and quantified using NanoDrop Fluorospectrometer ND3300 fragment size (Agilent Bioanalyzer 2100) prior sequencing. Followed by end repair and adaptor ligation and quantification of each library using Library Preparation kit (NEBNext® UltraT DNA Library Prep Kit for Illumina), before running them on illumina’s MiSeq platform following the amplicon sequencing protocol [24].

## Results

### Bacterial families and species identified in the remedies

**Fig. 2:**
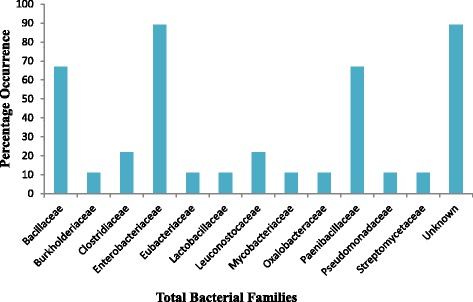
Percentage occurrence of each bacteria family identified in all the polyherbal remedy

The findings of this study revealed the presence of both pathogenic and non-pathogenic bacteria in the polyherbal medicines used for the treatment of tuberculosis in the Eastern Cape Province of South Africa. All the identified bacteria in the therapies belong to 12 families. Only one of the families could not be identified (Fig. 2). However, the prevalent families in all the polyherbal medicines are the Enterobacteriaceae, Bacillaceae and Paenibacillaceae (Fig. 2).

**Fig. 3:**
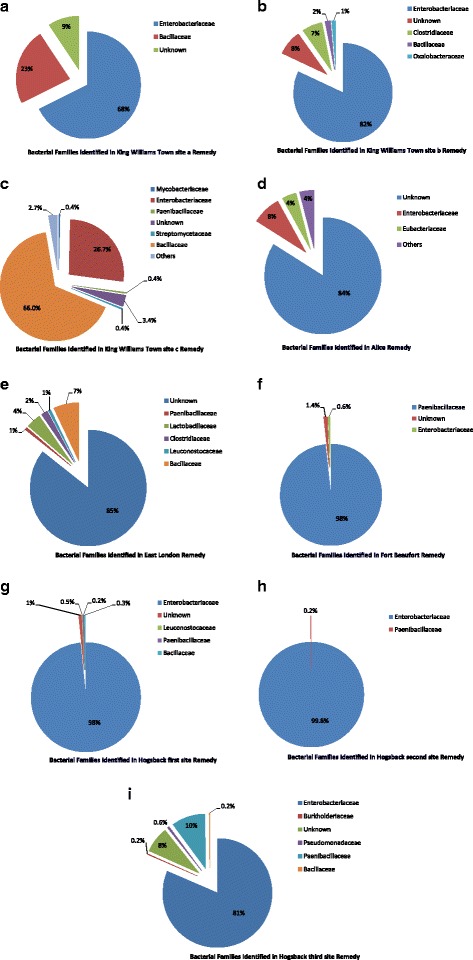
Relative frequencies of contaminating organisms in each polyherbal remedy

While the majority of the bacteria identified in KWTa remedy are Enterobacteriaceae (68 %), others were Bacillaceae (23 %), with 9 % remaining unknown (Fig. 3a). In the same vein, Enterobacteriaceae are the dominant organisms in KWTb remedy and only 8 % remaining unknown (Fig. 3b). The family Bacillaceae dominate KWTc therapy (66 %), whereas only 3 % of the bacteria are unknown (Fig. 3c). The identities of the majority of bacteria present in the herbal medicines sourced from AL and EL were unknown (84 and 86 %, respectively), with the remaining bacteria belonging to 16 and 5 families in AL and EL samples, respectively (Figs. 3d, e). A high number of bacteria identified in FB belong to Paenibacillaceae (98 %) as against a few other remedies where their population is insignificant (Fig. 3f). Similarly, HBfs, HBss and HBts remedies were dominated by Enterobacteriaceae (Figs. 3g–i).

This study further investigated the bacterial species present in each of the polyherbal medicines. The overall blast output revealed that these polyherbal remedies are contaminated with different bacterial genera and species including *Bacillus* sp., *Klebsiella* sp., *Rahnella* sp., *Paenibacillus* sp., *Enterobacter* sp., *Pantoea* sp., *Clostridium* sp., and a few unknown (Table 1). Some of these bacteria that are clinically pathogenic to human include *Raoultella ornithinolytica*, *Rahnella aquatilis*, *Bacillus anthracis*, *Bacillus cereus*, *Salmonella enteric*, *Enterobacter cloacae*, *Klebsiella oxytoca* and *Klebsiella pneumonia*. Others such as *Enterobacter asburiae*, *Paenibacillus polymyxa*, *Pantoea rwandensis*, *Klebsiella variicola* and *Pseudomonas* sp. are opportunistic pathogens causing opportunistic infections in individuals with impaired immunity.

**Table 1:** Pathogenic and non-pathogenic bacterial species identified metagenomically from the polyherbal medicines used for the treatment of tuberculosis in the Eastern Cape Province of South Africa

KWTa	KWTb	KWTc	AL	EL	FB	HBfs	HBss	HBts
*^a^ *Raoultella ornithinolytica*	*^a^ *Rahnella aquatilis*	^nb^ *Bacillus thuringiensis*	*^a^ *Rahnella aquatilis*	*^b^ *Bacillus cereus*	^h^ *Paenibacillus* sp.	^a^ *Enterobacter* sp.	*^a^ *Klebsiella oxytoca*	^nb^ *Bacillus subtilis*
* ^b^ *Bacillus anthracis*	^na^ *Klebsiella variicola*	* ^a^ *Rahnella aquatilis*	^nj^ *Eubacterium moniliforme*	^nb^ *Lysinibacillus sphaericus*	^nh^ *Paenibacillus terrae*	^h^ *Paenibacillus* sp.	^a^ *Enterobacter* sp.	^nh^ *Paenibacillus polymyxa*
*^b^ *Bacillus cereus*	*^a^ *Klebsiella oxytoca*	^b^ *Bacillus* sp.	Uncultured firmicutes	^nk^ *Lactobacillus* sp.	^a^ *Enterobacter* sp.	^na^ *Pectobacterium carotovorum*	^a^Uncultured enterobacter	^na^ *Pantoea rwandensis*
*^a^ *Klebsiella pneumoniae*	*^b^ *Bacillus cereus*	*^b^ *Bacillus cereus*	Uncultured bacterium	^ne^ *Clostridium thiosulfatireducens*	Uncultured bacterium	^b^ *Bacillus* sp.	^na^ *Enterobacter cloacae*	^na^ *Pantoea agglomerans*
*^a^ *Rahnella aquatilis*	*^a^ *Klebsiella pneumoniae*	Uncultured bacterium	–	^ne^ *Clostridium xylanolyticum*	Uncultured actinobacterium	^nl^ *Weissella soli*	^nh^ *Paenibacillus polymyxa*	*^a^ *Klebsiella pneumoniae*
^a^ *Enterobacter* sp.	*^a^ *Salmonella enterica*	^na^ *Kluyvera intermedia*	–	^h^ *Paenibacillus* sp.	–	Uncultured firmicutes	^na^ *Citrobacter freundii*	^nm^ *Burkholderia xenovorans*
^na^ *Klebsiella variicola*	^nf^ *Herbaspirillum frisingense*	^ng^ *Mycobacterium chelonae*	–	^e^Uncultured clostridium	–	Uncultured bacterium	–	^np^ *Pseudomonas* sp.
^na^ *Leclercia* sp.	^na^ *Enterobacter cloacae*	^nh^ *Paenibacillus* sp.	–	^nl^ *Weissella soli*	–	–	–	^na^ *Pantoea vagans*
^b^Uncultured bacillus	^na^ *Enterobacter asburiae*	^ni^ *Streptomyces leeuwenhoekii*	–	Uncultured bacterium	–	–	–	^a^ *Klebsiella* sp.
^c^ *Bacterium nxked5*	^e^Uncultured clostridium	–	–	^nl^ *Leuconostoc mesenteroides*	–	–	–	^a^ *Pantoea* sp.
^nb^ *Bacillus thuringiensis*	^e^ *Clostridium* sp.	–	–	^b^Uncultured bacilli	–	–	–	Uncultured bacterium
Uncultured bacterium	^c^ *Bacterium mj07*	–	–	–	–	–	–	–
–	^na^ *Kosakonia radicincitans*	–	–	–	–	–	–	–
–	^c^ *Bacterium mj15*	–	–	–	–	–	–	–
–	^c^ *Bacterium bx4*	–	–	–	–	–	–	–
–	Uncultured bacterium	–	–	–	–	–	–	–

Polyherbal medicines were collected from the following: *KWTa* King Williamstown site A, *KWTb* King Williamstown site B, *KWTc* King Williamstown site C, *AL* Alice, *EL* East London, *FB* Fort Beaufort, *HBft* Hogsback first treatment, *HBst* Hogsback second treatment, *HBtt* Hogsback third treatment

– absent, *pathogenic to human, *a* Enterobacteriaceae, *b* Bacilliaceae, *n* non-pathogenic, *c* unclassified bacteria, *e* Clostridiaceae, *f* Oxalobacteraceae, *g* Mycobacteriaceae, *h* Paenibacillaceae, *i* Streptomycetaceae, *j* Eubacteriaceae, *k* Lactobacillaceae, *l* Leuconostocaceae, *m* Burkholderiaceae, *p* Pseudomonadaceae

## Discussion

The use of polyherbal medicines for the treatment of various diseases is still a significant practice in the developing countries including South Africa. With the popularity and global market expansion, the safety of herbal products has become a major concern to public health [25]. In this study, all the polyherbal therapies used for the treatment of tuberculosis are orally consumed in the form of water-extracted remedies. Naturally, the ingredients used for the preparation of these remedies are not usually sterilized before soaking in water, hence the presence of different bacteria species and families identified in the polyherbal medicines. The main source of contamination could be from the soil, water, plant or other raw materials and the containers used. Since these remedies are not prepared in a sterile manner, another possible source of contamination could be contaminants from the personnel(s), unhygienic production conditions, during harvesting, drying and storage. In addition, environmental factors such as temperature, humidity and extent of rainfall during pre-harvesting and post-harvesting periods can influence the microbial contamination of these medicinal herbal [26].

The presence of Bacillaceae, such as *B. cereus* and *B. anthracis*, and Enterobacteriaceae including *R. ornithinolytica*, *Rahnella* sp., *Klebsiella* sp. and *Enterobacter* sp. in these polyherbal medicines is a cause for concern. Some of these bacteria are pathogenic to humans while others are opportunistic pathogens (Table 1) but could be serious public health hazard causing opportunistic infection and reduces the immunity of the immune-suppressed consumers. *R. ornithinolytica* (formerly named *Klebsiella ornithinolytica*) was identified in KWTa remedy. This bacterium is Gram-negative, non-motile, encapsulated and aerobic bacillus [27]. Though an uncommon human pathogen, about 86 infectious cases of this organism have been reported [28]. This pathogen has been linked to bacteremia [29, 30], sepsis [31], acute suppuration of the pancreatic duct [32], soft tissue infection [33], enteric fever [34], renal cysts [35] and urinary tract infection [36]. Also, it expresses chromosomal class A β-lactamases, which confer resistance to ampicillin and other aminopenicillins [28].

*R. aquatilis* was identified in AL remedy, KWTa, KWTb and KWTc remedies (Table 1). The bacterium is a facultatively anaerobic, nitrogen-fixing and Gram-negative rod-shaped organism. It has been reported to cause infections such as bacteremia, sepsis, respiratory infection, urinary tract infection and wound infections most especially in immune-suppressed individuals [37, 38]. Also, about 18 cases of human infection caused by *R. aquatilis* has been reported, and majority of these infections were accompanied by diabetes mellitus, alcoholism, cancer and AIDS [38]. The presence of this bacterium in the polyherbal remedies is a cause for concern considering the immunocompromised status of the patients.

*Bacillus* species such as *Bacillus cereus*, *B. anthracis* and *B. subtilis* were also identified in the polyherbal remedies. *Bacillus cereus* is a Gram-positive, aerobic-to-facultative, spore-forming rod bacterium bearing close phenotypic and genetic relationships to several *Bacillus* species most especially *B. anthracis* [39]. This bacterium is an emerging human food-borne pathogen. *B. cereus* has been reported to be associated with severe local and systemic human infections such as endophthalmitis, pneumonia, lung infections, bloody diarrhoea, gastroenteritis and meningitis, posing a serious public health problem [40, 41]. The pathogenicity of *B. cereus* depends on its ability to colonise, persist and subsequently invade the host tissues [42]. The ability of this bacterium to produce emetic toxins has been also associated with gastro- and non-gastrointestinal infections [43].

*B. anthracis* is an obligate pathogen that infects many vertebrates. It is the causative agent of anthrax. Anthrax is an infectious disease that can infect humans in three different ways, namely cutaneous anthrax, inhalation anthrax and gastrointestinal anthrax [44]. The presence of this bacterium in the remedies could be due to the availability of its endospores in the soil where the herb ingredients are harvested. The presence of *Bacillus* sp. in these remedies poses great risks to the consumers.

*Klebsiella* species are Gram-negative, non-motile and usually encapsulated rod-shaped bacteria [45]. Species of this genus have been increasingly associated with hospital infections [46]. They are common pathogens of nosocomial pneumonia, septicaemia, urinary tract infection, wound infections, intensive care unit infections and neonatal septicaemias [45]. *K. pneumoniae*, *K. oxytoca* and *K. variicola* were detected in the remedies. *K. pneumoniae* is the most pathogenic to humans. Infections with this bacterium are usually hospital-acquired. These include urinary tract infection, pneumonia, intra-abdominal infection, bloodstream infection, meningitis and pyogenic liver abscess [47]. *K. pneumoniae* has been reported to posses several intrinsic and acquired mechanisms which make it resistant to several antimicrobial agents [48, 49].

*S. enterica* was identified in KWTb remedy. It is a rod-shaped, Gram-negative, flagellated facultative anaerobe. The bacterium is a medically important pathogen of both humans and animals. The presence of this bacterium in this remedy is of concern because *S. enterica* is known to cause diseases such as gastroenteritis, septicaemia and enteric fever clinically [50]. Like many other infectious diseases, the infection severity of *Salmonella* may vary depending on the resistance of individual to the pathogen, the immune system and the virulence strain [50].

*Enterobacter* species are rod-shaped, non-spore-forming, facultative anaerobes and Gram-negative bacilli bacteria. Several strains of these bacteria are increasingly been identified as nosocomial pathogens causing infections in hosts with impaired immunity [51]. They have been reported to cause 5 % of hospital-acquired septicemias, 5 % of nosocomial pneumonias, 4 % of nosocomial urinary tract infections and 10 % of postsurgical peritonitis cases [52]. Skin and soft tissue infection, endocarditis, intra-abdominal infection, septic arthritis, osteomyelitis and ophthalmic infection have been associated with these bacteria. *E. cloacae* is ubiquitous in nature, although generally not known to be an enteric pathogen; it is an opportunistic pathogen in humans [53, 54]. A wide spectrum of infections such as the urinary tract, lower respiratory tract, skin and soft tissue, biliary tract, wounds, intravenous catheters and the central nervous system have been associated with *Enterobacter cloacae* [55].

None of the analysed marketed polyherbal remedies had any form of food-based tests carried out on them since they are locally made medicines. This may probably account for the high discovery of bacterial population in the remedies. The results from different findings conducted on microbial quality of traditional herbal medicines have been alarming revealing the presence of pathogenic bacteria and other contaminants in the herbal therapies [13–15, 17, 56]. Several fatal infectious outbreaks have been associated with the use of heavily contaminated raw materials of natural origin with pathogens; thus, great efforts are necessary to guarantee constant and adequate quality [57].

The prevalence of the members of Enterobacteriaceae in all the herbal remedies was recorded (Fig. 2). Enterobacteria are found in nature and are often thought as indicating faecal contamination. Thus, their presence could be regarded as an index of the degree of contamination, which may indicate possible presence of harmful or disease causing organisms [58–60]. Also, higher numbers of spore-forming bacteria such as *Bacillus* sp. and a few numbers of *Clostridium* sp. found in the remedies could be due to the fact that some of these organisms produce spores which are resistant to harsh processing, elevated heat and dry conditions, thereby able to survive for a very long time on the products in dormant states [26]. Likewise, the members of Paenibacillaceae which are Gram-positive and are aerobic bacteria that are related to *Bacilli* were found in the remedies. Until recently, these organisms were not known to cause human disease, but now, there are several reports of human infections caused by some members of this genus [61].

## Conclusions

The findings of this study revealed different bacteria populations in the polyherbal medicines used for the treatment of tuberculosis in the Eastern Cape Province of South Africa; this is a cause for concern. Since there are no legislative criteria governing the microbial quality of these therapies in South Africa, there is an urgent need for the Government to take adequate control measures to set specific standards for quality of these medicines, considering the fact that these medicines are being taking by immunocompromised individuals. Also, there is a need to educate the public as well as the traditional healers in the Province, that proper hygienic condition should be maintained in all preparation processes starting from plant collection, processing, packaging and storage, as well as emphasizing on the implications of non-compliance to hygienic conditions to both the consumers and the traditional healers.
